# Forecasting the Impact of Climate Change on *Apis dorsata* (Fabricius, 1793) Habitat and Distribution in Pakistan

**DOI:** 10.3390/insects16030289

**Published:** 2025-03-11

**Authors:** Tauheed Ullah Khan, Xiaofeng Luan, Ghulam Nabi, Muhammad Fahad Raza, Arshad Iqbal, Shahid Niaz Khan, Huijian Hu

**Affiliations:** 1 Guangdong Key Laboratory of Animal Conservation and Resource Utilization, Institute of Zoology, Guangdong Academy of Sciences, Guangzhou 510260, Chinafbiuaf91@gmail.com (M.F.R.); 2 School of Ecology and Nature Conservation, Beijing Forestry University, Beijing 100083, China; 3 Department of Zoology, Institute of Molecular Biology and Biotechnology, University of Lahore, Lahore 54000, Pakistan; 4 Center of Biotechnology and Microbiology, University of Swat, Swat 19120, Pakistan; 5 Department of Zoology, Kohat University of Science and Technology, Kohat 26000, Pakistan

**Keywords:** *Apis dorsata*, climate change, current habitat, future distribution, projected loss, conservation, Pakistan

## Abstract

Climate change has led to global biodiversity loss, severely impacting all species, including essential pollinators like bees, which are highly sensitive to environmental changes. Like other bee species, *A. dorsata* is also not immune to climate change. This study evaluated the habitat suitability of *A. dorsata* under climate change in Pakistan by utilizing two years of occurrence and distribution data to develop a Maximum Entropy (MaxEnt) model for forecasting current and future habitat distribution. Future habitat projections for 2050 and 2070 were based on two shared socioeconomic pathways (SSP245 and SSP585) using the CNRM-CM6-1 and EPI-ESM1-2-HR-1 global circulation models. Eight bioclimatic variables (Bio1, Bio4, Bio5, Bio8, Bio10, Bio12, Bio18, and Bio19) were selected for modeling, and among the selected variables, the mean temperature of the wettest quarter (Bio8) and precipitation of the warmest quarter (Bio18) showed major contributions to the model building and strongest influence on habitat of *A. dorsata*. The model estimated 23% of our study area as a suitable habitat for *A. dorsata* under current climatic conditions, comprising 150,975 km^2^ of moderately suitable and 49,792 km^2^ of highly suitable regions. For future climatic scenarios, our model projected significant habitat loss for *A. dorsata* with a shrinkage and shift towards northern, higher-altitude regions, particularly in Khyber Pakhtunkhwa and the Himalayan foothills. Habitat projections under the extreme climatic scenario (SSP585) are particularly alarming, indicating a substantial loss of the suitable habitat for the *A. dorsata* of 40% under CNRM-CM6-1 and 79% for EPI-ESM1-2-HR-1 for the 2070 time period. This study emphasizes the critical need for conservation efforts to protect *A. dorsata* and highlights the species’ role in pollination and supporting the apiculture industry in Pakistan.

## Simple Summary

Climate change is threatening pollinators like *Apis dorsata* (the giant honey bee), which are vital for ecosystems and agriculture. This study mapped the current habitat and future distribution of *A. dorsata* in Pakistan using field surveys and climate models. Currently, 23% of our study area is suitable for the species, but future climate scenarios predict a significant loss of habitat, especially in northern high-altitude regions. By 2061–2080, up to 79% of suitable habitat could disappear under extreme climate conditions. These findings highlight the urgent need for conservation efforts to protect *A. dorsata* and the beekeeping industry in Pakistan.

## 1. Introduction

Climate change is affecting every ecosystem [1,2,3,4] and has caused a substantial biodiversity loss [4,5,6]. It has significantly impacted species habitats [7,8] at various local and global geographical scales, leading to habitat degradation and biodiversity loss [9,10,11,12,13]. Habitat degradation driven by climate change is a leading cause for species declines and extinctions [14,15,16,17]. By reducing the available and distribution of suitable habitats [18,19,20,21,22], climate change forces species into less favorable environments [22], severely compromising their survival [4,15,23,24,25,26,27]. These shifts in habitat distribution threaten the survival of diverse life-forms, including plants [28], animals [29], and insects and pollinators [30,31,32,33,34,35]. Pollinators, such as honey bees, are particularly vulnerable due to their reliance on stable environmental conditions and floral resources.

Through pollination, honey bees play a critical role in sustaining global ecosystems [36,37,38]. Therefore, the survival and health of honey bees are essential for agriculture and biodiversity conservation [39,40,41]. Beekeeping also brings economic benefits to rural areas in developing countries like Pakistan, thus alleviating poverty [42]. Honey bees pollinate and make honey and royal jelly, which are used as food and play an essential role in medicines and cosmetics. Other major bee products like bee venom, propolis, pollen, and wax are also very important economically and used in pharmaceutical industries [43,44,45]. Honey bees are of economic importance to humans because they produce honey in substantial amounts compared to other honey bee species. They can yield between 50 and 80 kg of honey per colony, as recorded by [46]. However, wild bee species, such as *Apis dorsata*, are not domesticated and play a more significant role in pollination than in honey production [47]. Unfortunately, the honey bee population globally faces several challenges, including exposure to chemical pollution [48], emerging pathogens [39] climate change [49], and escalating anthropogenic activities [50,51]. Therefore, understanding the climatic and environmental impacts on honey bee survival and health is essential for conservation and management. Climate change is depleting bee habitats and food supplies, resulting in severe bee population declines [51]. In comparison to managed bees, wild bees are considerably more challenging to conserve due to their limited control over their livelihood and movement pattern [52].

Among the wild honey bees in Pakistan, *Apis dorsata* (Fabricius, 1793) is one of the most ecologically significant species [53]. *A. dorsata*, also called the rock honey bee, inhibits the plains and foothills of Pakistan. In mountainous regions, such as the Himalayan highlands, it can be found at 1000 to 1700 m above sea level, and they can even migrate up to 2000 m during seasonal migrations to find new nesting sites or food sources [54]. It frequently builds nests in the air, between 3 and 25 m above ground [55]. Combs of *A. dorsata* are typically large, measuring 1.5 to 2.1 m wide and 0.6 to 1.2 m tall, which makes them unique among wild bees [56]. It has adapted to regions in Pakistan characterized by tall trees and hilly terrains. A study on diversity and abundance of honey bee species conducted in Murree (the northern mountainous region of Pakistan) [57] concluded that *A. dorsata* is the most prevalent species in the area. This species is also reported in Rawalpindi, Khaniwal, Lahore, Kasur, Chakwal, Nankana, Attock, Narowal, Haripur, Jhelum, Gujrat, Sialkot, (Punjab), Mirpur, and Bhimber (AJK) [58,59,60].

Species distribution modeling (SDM) offers a valuable tool for studying how environmental conditions and climatic factors influence species habitat and distribution. Many SDMs have been developed for estimating the relationship between environmental factors and species occurrence records and for predicting potential distribution of species, including Maximum Entropy [61], ecological niche factor analysis, Climex Dymex, and bioclimatic modeling. Among these, MaxEnt is one of the most popular methods since it has high precision and strong robustness [62,63,64,65,66,67,68,69,70,71]. Maximum Entropy (MaxEnt) can assess both continuous and categorical data and produce a readily interpretable continuous probabilistic output by using a small sample size with high accuracy and statistical significance.

In Pakistan, agriculture is a cornerstone of the economy, and pollinators like *A. dorsata* (rock honey bee) are critical for the pollination of cash crops such as fruits, vegetables, and oilseeds. These crops have a combined pollination-dependent production value of USD 1.59 billion, underscoring the economic and nutritional importance of pollinators [72]. However, climate change, deforestation, excessive pesticide use, and other anthropogenic pressures are threatening pollinator populations, contributing to habitat loss and food insecurity [73,74]. Ranked fifth among the most climate-vulnerable nations, Pakistan is highly susceptible to the impacts of climate change [75,76]. Rising annual average temperatures, unprecedented rainfall, glacier shrinkage, and frequent flooding are affecting species and ecosystems across the country [77,78,79,80,81,82,83], contributing to biodiversity loss and species-suitable habitats [71,84,85]. Therefore, this study seeks to assess the effects of rising temperatures and related environmental changes on the habitat suitability of *A. dorsata* in Pakistan. It is hypothesized that climate-induced temperature increases and environmental shifts will lead to a decline in suitable habitats for *A. dorsata*.

## 2. Materials and Methods

### 2.1. Study Area

**Figure 1:**
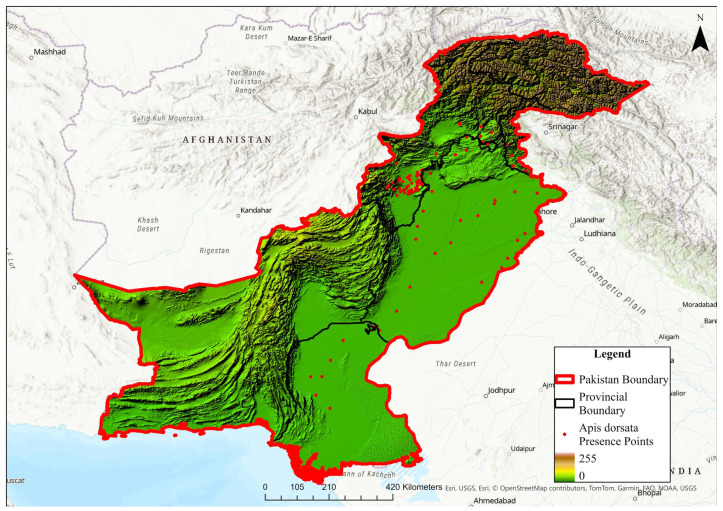
Map of the study area and distribution of the *A. dorsata* occurrence used for habitat suitability modeling under current and future climatic conditions. The color gradient represents elevation, ranging from lowland areas (green) to higher elevations (light brown to dark brown).

Pakistan is geographically very diverse, with the Arabian Sea in the south and the world’s second-highest mountain peak (K2) in the north [86]. The study area lies between 24° and 37° north and 61° and 75° east, over an area of 458,383 km^2^ (Figure 1). Stretching from the Indian Ocean’s southern coastline to the northern mountain ranges, which rise to an elevation of 7289 m, it encompasses a diverse landscape [87]. The research area’s diversified terrain supports a rich array of biodiversity.

### 2.2. Occurrence/Presence Data

Field surveys were conducted from 2021 to 2022 across diverse habitats and environmental conditions within the study area to collect data on the presence and distribution of *A. dorsata*. We conducted questionnaire surveys and interviews with field visits to obtain specific input from local communities, including wildlife experts, residents, and bee keepers. Informed consent was obtained from all study participants before conducting interviews. A questionnaire survey provides reliable information about species status in a particular area [88,89,90]. Furthermore, the previous literature on different ecological aspects of *A. dorsata* in Pakistan was examined since it offers reliable information about the species distribution and occurrence [91]. Google Earth (http://ditu.google.cn/, accessed on 1 October 2024) was used to ascertain coordinates for presence points extracted from the list of the literature (Supplementary Material Table S1). Additionally, we searched international repositories, including GBIF (Global Biodiversity Information Facility), VertNet, and BIEN (Botanical Information and Ecology Network), to validate and complement our field-collected data. However, no relevant presence points for *A. dorsata* were found in these datasets. To ensure robust data collection, we cross-verified presence points from three sources: questionnaire interviews, the literature [59,60,92,93,94,95,96,97,98,99,100,101,102,103,104,105,106,107], and field surveys. Field survey points were prioritized for their reliability and accuracy. This meticulous method enhanced data quality and leveraged multiple sources to strengthen the study’s findings.

### 2.3. Thinning and Partition Occurrence Data Preprocessing of Occurrence/Presence Data

Outliers and duplicate points were removed to reduce the influence of redundancy on model predictions. We obtained a total of 318 occurrence points during the data collection phase. Using the R (version 4.4.1, R Core Team, Vienna, Austria, 2024) package *spThin*, localities less than 5 km apart were filtered out, resulting in a final dataset of 91 occurrence points used for modeling [108]. Finally, the MaxEnt model employed the rarefied occurrence points’ coordinates (projection WGS 1984). Researchers often partition a single biodiversity dataset into subsets to evaluate the accuracy of a predictive model. Truly independent biodiversity data are often difficult to obtain, so researchers typically validate predictive models using subsets of the same dataset. Occurrence data are divided into two categories: (1) training or calibration data for model development, and (2) testing or evaluation data for model validation [109,110]. We used the R package *ENMeval* to partition occurrence localities into training and testing datasets. A Jackknife procedure was applied, assigning each occurrence record to a unique group (k = number of localities), a method well-suited for small sample sizes [111,112].

### 2.4. Selection and Processing of Environmental Variables

Algorithms for niche/distributional modeling need environmental predictor factors and occurrence data [113,114]. In recent years, several global databases of climatic data have emerged [115,116,117,118]. We used the R package geodata [119] to download WorldClim v2.1 (www.worldclim.org) (accessed on 5 October 2024). A total of 19 bioclimatic variables (Supplementary Material Table S2), representing different features of temperature and precipitation, were developed by spatially interpolating monthly data from meteorological stations using elevation as a covariate. The variables in habitat suitability models were carefully chosen due to their significance in influencing distributions of species [120]. WorldClim bioclimatic variables are key to defining species niches [121,122] and are widely applied in species distribution modeling [24,120,122,123]. The bioclimatic variables used in our study, for both the current period and future scenarios, were obtained at a spatial resolution of 2.5 arc minutes.

**Figure 2:**
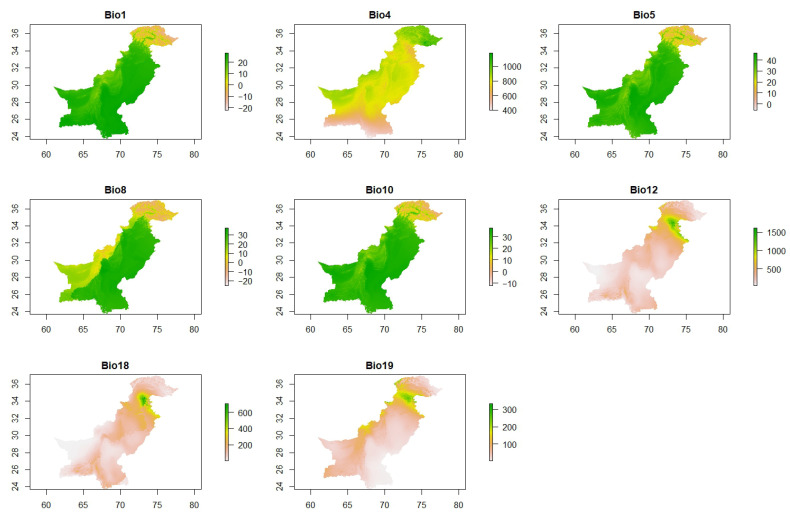
Selected variables with less than 0.7 correlation used for model building. The variables shown in the plots include Bio1 (annual mean temperature), Bio4 (temperature seasonality, standard deviation × 100), Bio5 (maximum temperature of the warmest month), Bio8 (mean temperature of the wettest quarter), Bio10 (mean temperature of the warmest quarter), Bio12 (annual precipitation), Bio18 (precipitation of the warmest quarter), and Bio19 (precipitation of the coldest quarter). Each plot represents the geographic distribution of the respective variable across the study area, with the axes indicating latitude (*y*-axis) and longitude (*x*-axis).

To ensure data consistency, bioclimatic variables were standardized and aligned to a common coordinate system and resolution. To minimize the risk of model overfitting, variables with minimal contribution (e.g., percentage contributions close to 0 or below 0.25) identified through Jackknife testing were excluded, following guidance from previous studies [124,125]. Given that several bioclimatic variables exhibited spatial correlation, further collinearity tests were conducted to avoid overfitting from highly correlated variables [125,126]. A Pearson Correlation Coefficient threshold of |r| ≥ 0.75 [127] (Supplementary Material Figure S1) was applied to identify and eliminate variables with strong correlations and enhance the model’s predictive performance. Ultimately, eight bioclimatic variables (Figure 2) that exhibited low correlation (|r| < 0.75) were retained for habitat suitability modeling [127]. The selected variables used for modeling are presented in Figure 2.

### 2.5. Construction of Maximum Entropy (MaxEnt) Model

This study used MaxEnt (v. 3.4.3) [128,129] to identify the suitable habitat available for *A. dorsata* in the study area. It is an efficient model [62,63,64,65] to assess the potential habitat and distribution of a species in an area [64,85,126,130]. Furthermore, it is particularly suitable when occurrence data consist only of presence points [111,126,131,132,133,134,135], as accurately obtaining absence data remains challenging [64,126,136,137]. It is also effective for small sample sizes [134]. This module utilizes the R packages *ENMeval* and *dismo* [119,138] to build and evaluate MaxEnt niche models across various feature class settings and regularization multipliers [139].

### 2.6. Model Evaluation and Selection Process

There are several metrics to assess the performance of niche/distributional models. We conducted a systematic tuning process with modifications in feature classes and regularization multipliers to find an optimized configuration of the model that projects *A. dorsata* habitat suitability using MaxEnt. We ran four feature classes, which are Linear (L), Linear and Quadratic (LQ), Linear, Quadratic, and Hinge (LQH), and Linear, Quadratic, Hinge, and Product (LQHP) for three values for RM: 0.5, 1, and 1.5. Model performance was assessed based on Delta AICc, Akaike weights, Average Validation AUC, and model complexity (measured by the number of parameters). Additionally, following the recommendations of [140], we evaluated test-training AUC differences and omission rates to ensure robust model performance and avoid overfitting. The Jackknife test [64,141,142] was used to determine the percent contribution and relative importance of predictor variables for *A. dorsata* habitat suitability and distribution, with *ENMeval* 2.0 [138] facilitating the analysis.

### 2.7. Future Projection Data

We used data from two global climate models, CNRM-CM6-1 and EPI-ESM1-2-HR-1, to simulate future projections of habitat suitability for *A. dorsata*. The models correspond to two socioeconomic pathways, SSP245 and SSP585, representing moderate and extreme climate scenarios, respectively. These pathways represent distinct climatic futures shaped by varying greenhouse emissions and socioeconomic developments, making them valuable for assessing potential habitat changes in the study area. We used the functionality of model prediction grids from the R package *dismo* [143] and *geodata* for accessing climate data [119]. The CNRM-CM6-1 model, developed by France’s National Center for Meteorological Research, was chosen for its ability to simulate South Asian monsoons and regional climate patterns, both critical for *A. dorsata* habitats. The EPI-ESM1-2-HR-1 model, from the Institute of Atmospheric Physics in China, provides high-resolution climate simulations that aligns with Pakistan’s diverse topography and climate zones. These models’ regional accuracies make them ideal for studying future habitat projections in Pakistan. Future habitat projections were generated for two time periods: 2041–2060 (2050) and 2061–2080 (2070), using the SSP245 and SSP585 scenarios. These models allow comparisons between moderate and extreme climate futures. The high spatial and temporal resolution of the models ensured accurate predictions of habitat suitability across the study area.

### 2.8. Division of Potential Suitable Growing Areas for Apis dorsata

The Jenks natural breaks classification technique in GIS was used to reclassify the model simulation results (raster layer). The raster was reclassified using this method by minimizing differences within categories and classes, while increasing differences between them. Based on several studies, this study reclassified the potential species habitat into four classes, including highly suitable (≥0.71), moderately suitable (0.31–0.70), less suitable (0.11–0.30), and unsuitable habitats (≤0.10) [28,71,144].

## 3. Results

### 3.1. Model Performance and Selection

**Figure 3:**
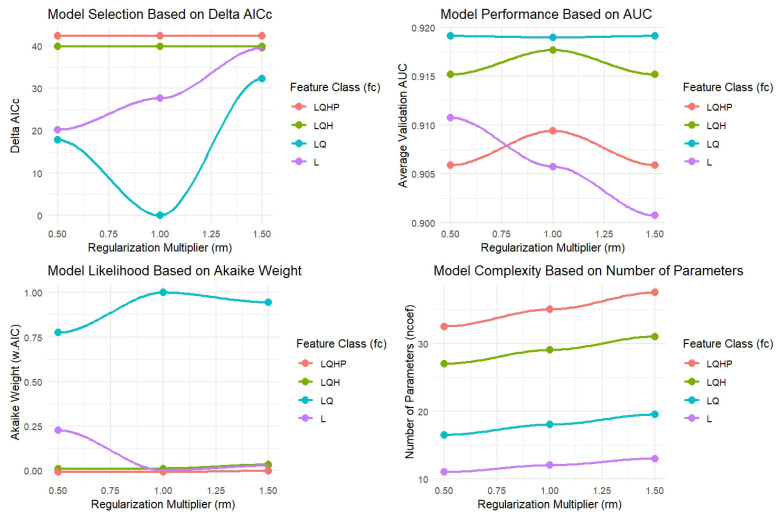
Comparative evaluation of model performance metrics for different feature classes (L, LQ, LQH, and LQHP) across a range of regularization multipliers (rms).

Among the tested configurations, LQ with RM:1 emerged as the best-performing model (Figure 3). This configuration had the lowest Delta AICc, indicating a strong model fit with minimal complexity. Additionally, it achieved the highest AUC score (0.91) and a training AUC of 0.94, showing minimal divergence between training and validation datasets, which confirms the model’s robustness. The 10th percentile omission rate was calculated as 0.07, which is well below the commonly accepted threshold of 0.1, further validating the reliability of the selected model. These results, in combination with the Akaike weight (nearly 1.0), indicate that LQ with RM:1 was the optimal model configuration among those tested (LQ, LQH, and LQHP). Notably, LQ with RM:1 also maintained a moderate number of parameters, balancing model performance with interpretability. Based on these results, we selected LQ with RM:1 for current and future projections to ensure robust and generalizable habitat suitability predictions for *Apis dorsata*.

### 3.2. Response of A. dorsata Habitat Suitability to Key Bioclimatic Variables

**Figure 4:**
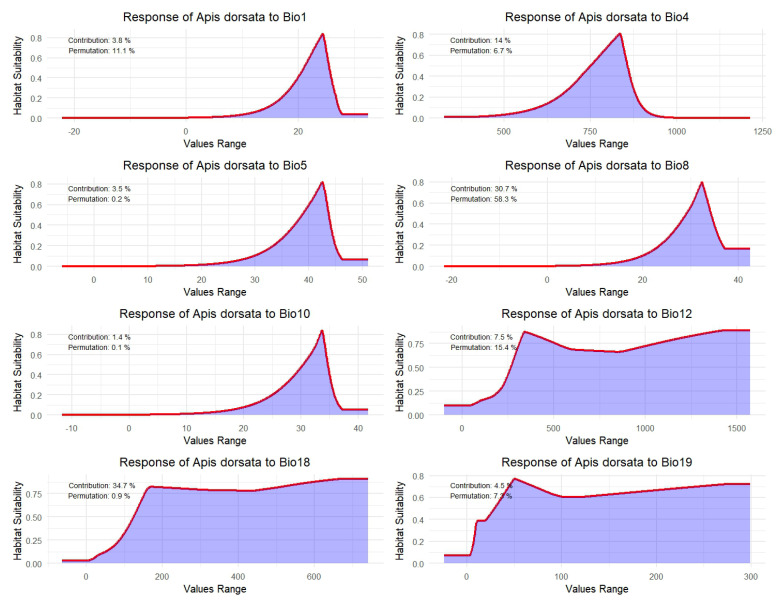
Response curves showing the habitat suitability of *A. dorsata* across the range of selected environmental variables (Bio1: annual mean temperature, Bio4: temperature seasonality, Bio5: maximum temperature of the warmest month, Bio8: mean temperature of the wettest quarter, Bio10: mean temperature of the warmest quarter, Bio12: annual precipitation, Bio18: precipitation of the warmest quarter, and Bio19: precipitation of the coldest quarter). The red line indicates the modeled response of habitat suitability, while the shaded area reflects the overall trend of suitability distribution. The values on each plot show the percentage contribution and permutation importance based on the Jackknife test, providing insight into the significance of each variable in model construction.

The response curves revealed that habitat suitability for *A. dorsata* generally increases with moderate temperature and precipitation levels but declines at extreme values (Figure 4). For annual mean temperature and temperature seasonality, suitability rises with moderate conditions and drops at higher variability. Similarly, suitability peaks at moderate maximum temperatures and decreases with excessively high temperatures, indicating the species’ preference for moderate warmth. Precipitation patterns showed that *A. dorsata* favors habitats with moderate annual and seasonal rainfall, with suitability stabilizing or declining at very high levels. These trends highlight the species’ adaptation to balanced, moderate climatic conditions across key temperature and precipitation variables, helping to refine its habitat requirements.

The estimated contribution and permutation importance provide a rank of the environmental variables based on their relative influence on model performance while predicting the habitat suitability of *A. dorsata*. Among these variables, the precipitation of the wettest quarter (Bio8) and the coldest quarter (Bio18) showed the largest contribution and therefore prove to be highly influential in shaping suitable habitats. Both temperature seasonality and annual mean temperature were important in the case of temperature-related variables, indicating the sensitivity of *A. dorsata* to both stable and moderate temperature conditions. Other variables like maximum temperature have small values from permutation importance, indicating that though they contribute to the accuracy of the models, they might have lower predictive value in their own right and hence indicate a joint effect of many variables towards robust modeling for habitat suitability.

### 3.3. Species Habitat Under Current Environmental Conditions

**Figure 5:**
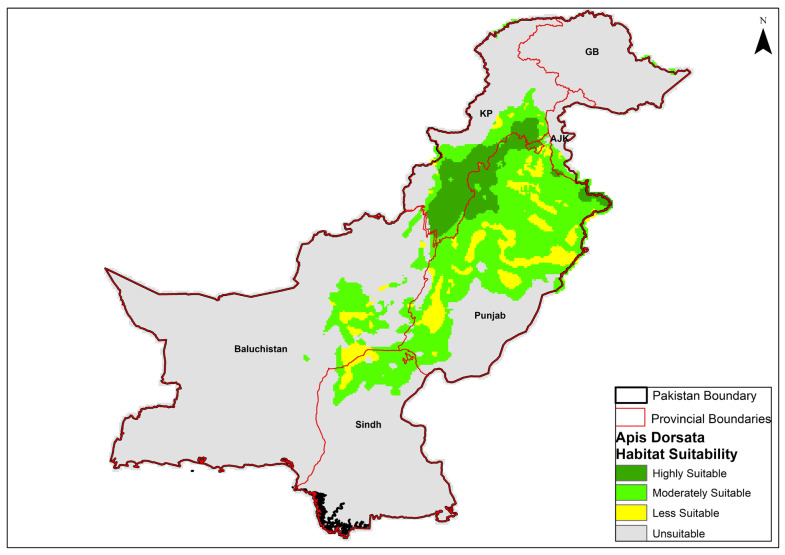
Current habitat suitability map for *Apis dorsata*. The abbreviations indicate the provinces and regions of Pakistan: GB (Gilgit Baltistan), AJK (Azad Jammu and Kashmir), KP (Khyber Pakhtunkhwa), Punjab, Sindh and Balochistan.

Under current climatic conditions, the habitat suitability model for *A. dorsata* showed significant variations across different provinces of Pakistan. The habitat was categorized into four classes including unsuitable, less suitable, moderately suitable, and highly suitable. The model predicts that highly suitable habitats span approximately 49,792 km^2^ (Table 1), primarily located in the Punjab and Khyber Pakhtunkhwa (KP) provinces (Figure 5). These regions, known for their diverse climatic conditions and moderate altitudes, provide optimal conditions for the species. Notable areas of high suitability extend into central Punjab and the southern parts of KP, encompassing districts such as Mianwali, Bhakkar, Isa Khel, Kalabagh, Fateh Jang, and Attock in Punjab and Dera Ismail Khan, Lakki Marwat, Bannu, Karak, Kohat, Charsadda, Nowshera, and Swabi in KP. Moderately suitable habitats cover about 150,975 km^2^ and are distributed more broadly. The central and southern regions of Punjab and parts of Sindh and northern Baluchistan contribute significantly to this category. These areas feature intermediate environmental conditions that support *A. dorsata* to a lesser extent than highly suitable areas. Less suitable habitats, totaling 51,312 km^2^, are scattered primarily around the periphery of moderately suitable regions, including southern Punjab and parts of eastern Sindh. The largest portion of the landscape comprising 629,817 km^2^ is deemed unsuitable for the species. This category dominates Baluchistan and extends to the extreme northern areas, including Gilgit-Baltistan (GB) and the Azad Jammu and Kashmir (AJK) regions, where climatic and environmental conditions fall outside the preferred range for *A. dorsata*.

### 3.4. Quantification and Distribution of Future Suitable Habitat of Apis dorsata

**Figure 6:**
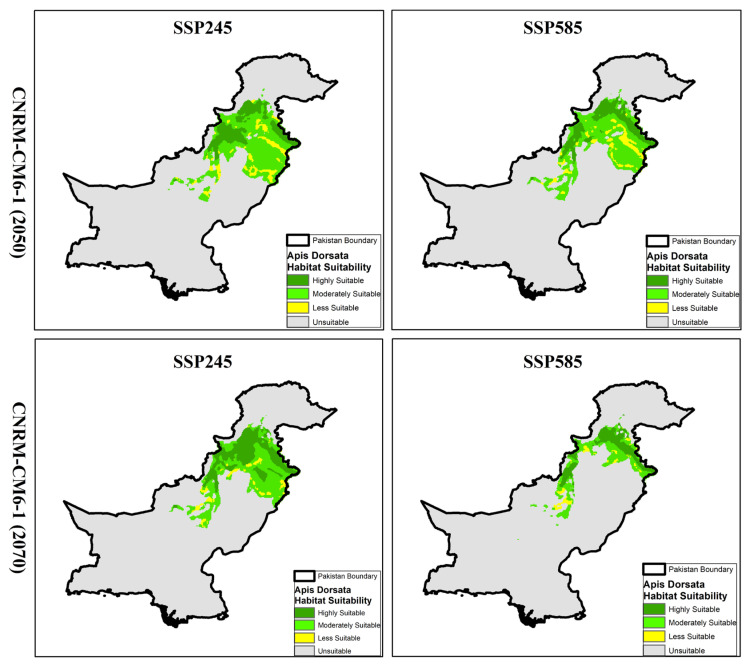
Projected distribution maps of *A. dorsata* habitat suitability in Pakistan under CNRM-CM6-1 model for SSP245 and SSP585 scenarios in mid-century (2050) and late-century (2070). Maps indicate shifts in suitable habitats with varying degrees of climate impact.

**Figure 7:**
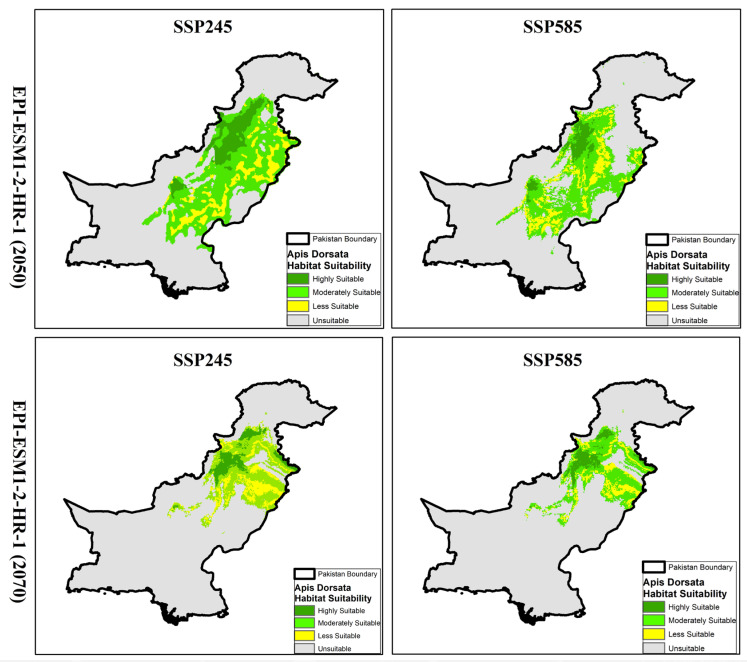
Projected distribution maps of *A. dorsata* habitat suitability in Pakistan under EPI-ESM1-2-HR-1 model for SSP245 and SSP585 scenarios in mid-century (2050) and late-century (2070). Maps indicate shifts in suitable habitats with varying degrees of climate impact.

Based on the future projections of *A. dorsata* habitat suitability under different climate models (CNRM-CM6-1 and EPI-ESM1-2-HR-1) and socioeconomic pathways (SSP245 and SSP585), the results showed a substantial loss and shift in suitable habitats across Pakistan by mid-century (2050) and late-century (2070). The distribution maps (Figure 6 and Figure 7) illustrate these changes, highlighting trends in habitat suitability under various greenhouse gas concentration pathways.

### 3.5. Habitat Projections and Distribution of A. dorsata Under CNRM-CM6-1 Model

Under future climate projections using the CNRM-CM6-1 model, significant changes in the distribution of *A. dorsata* habitats are anticipated by mid-century (2050) and late-century (2070) under both SSP245 and SSP585 scenarios. Compared to current conditions, where highly suitable habitats span approximately 49,792 km^2^, future projections showed a decline in these areas. For the mid-century under SSP245, highly suitable habitats decrease to 39,073 km^2^ and further decline to 36,011 km^2^ by late-century. Similarly, the SSP585 scenario forecasts an even steeper decline, with highly suitable habitats shrinking to 35,965 km^2^ by mid-century and 29,743 km^2^ by late-century. Moderately suitable areas, which currently cover about 150,975 km^2^, are also projected to decrease under future conditions. Mid-century estimates under SSP245 predict 103,135 km^2^ of moderately suitable habitats, reducing to 86,274 km^2^ by late-century. Under SSP585, these areas shrink from 93,156 km^2^ in the mid-century projection to 45,691 km^2^ in the late-century projection. These results suggest a pronounced shift and contraction of suitable habitats, indicating a potential movement of suitable habitat zones towards higher altitudes or more northern regions in response to changing climatic conditions. Expanding unsuitable and less suitable habitat categories also point to increasing challenges for *A. dorsata* habitat sustainability. For instance, unsuitable areas, currently covering 629,817 km^2^, are projected to grow significantly, reaching up to 792,177 km^2^ under SSP585 by late-century. This shift underscores the potential impacts of climate change on the habitat range, pushing *A. dorsata* towards regions that can sustain its ecological needs amidst changing conditions.

### 3.6. Habitat Projections and Distribution of A. dorsata Under EPI-ESM1-2-HR-1 Model

Based on future projections using the EPI-ESM1-2-HR-1 climate model, habitat suitability for *A. dorsata* demonstrated significant loss under different SSP scenarios for both mid-century (2050) and late-century (2070). In the SSP245 scenario for 2050, highly suitable habitats are predicted to cover approximately 27,865 km^2^, while moderately suitable areas span around 96,561 km^2^. Less suitable habitats account for 43,872 km^2^, and unsuitable areas dominate with 713,598 km^2^. This indicates a shift towards more habitats becoming unsuitable compared to current conditions. In the SSP585 scenario for the same period, the highly suitable habitat reduces further to 22,329 km^2^, with moderately suitable areas covering 86,756 km^2^. Less suitable regions decrease to 34,916 km^2^, and the extent of unsuitable habitats increases to 737,895 km^2^, showing a marked trend of habitat degradation under more intense greenhouse gas concentrations. Projections for 2070 under the SSP245 scenario reveal continued habitat reduction, with highly suitable areas decreasing to 24,547 km^2^ and moderately suitable areas remaining at 86,721 km^2^. Unsuitable habitats expand to 741,035 km^2^, further emphasizing the negative impact of climate change over time. The SSP585 scenario for 2070 showed the most severe impact, with highly suitable habitats reduced to just 10,646 km^2^ and moderately suitable areas shrinking to 54,756 km^2^. The extent of unsuitable habitats peak at 802,169 km^2^, signifying significant future habitat loss for *A. dorsata* under a high-emission scenario.

### 3.7. Percentage Change in Habitat Suitability Categories

**Figure 8:**
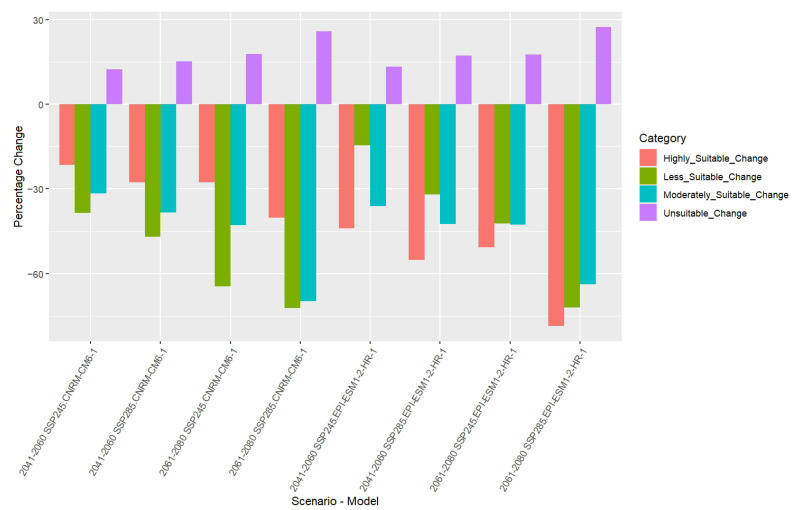
Percentage changes in habitat suitability categories for *A. dorsata* under future climate scenarios based on projections from CNRM-CM6-1 and EPI-ESM1-2-HR-1 models for SSP245 and SSP585 during mid-century (2050) and late-century (2070) projections.

Based on the percentage change in habitat suitability categories projected under different future scenarios, significant trends were observed (Table 2, Figure 8). For the CNRM-CM6-1 model during the 2050 period under SSP245, the unsuitable habitat showed a moderate increase of approximately 12.43%, while less suitable, moderately suitable, and highly suitable habitats exhibited notable declines of 38.48%, 31.69%, and 21.53%, respectively. Under SSP585 for the same period, the trend continued with a larger increase in unsuitable areas (15.20%) and more pronounced decreases in less suitable (47.00%), moderately suitable (38.30%), and highly suitable habitats (27.77%). By 2061–2080 under SSP245, the increase in the unsuitable habitat further intensified to 17.72%, accompanied by severe reductions in less suitable (64.60%), moderately suitable (42.86%), and highly suitable areas (27.68%). SSP585 projected an even greater shift, with the unsuitable habitat increasing by 25.78% and dramatic losses across the less suitable (72.16%), moderately suitable (69.74%), and highly suitable categories (40.27%).

For the EPI-ESM1-2-HR-1 model, the period 2050 under SSP245 showed an increase in the unsuitable habitat by 13.30%, while reductions were seen in less suitable (14.50%), moderately suitable (36.04%), and highly suitable habitats (44.04%). Under SSP585, the unsuitable habitat increased by 17.16%, and other categories saw more pronounced declines: less suitable (31.95%), moderately suitable (42.54%), and highly suitable (55.16%). During 2070, projections under SSP245 estimated a 17.66% rise in the unsuitable habitat, with declines in less suitable (42.33%), moderately suitable (42.56%), and highly suitable areas (50.70%). The SSP585 scenario projected the most drastic changes, with the unsuitable habitat increasing by 27.37% and significant reductions in less suitable (72.08%), moderately suitable (63.73%), and highly suitable areas (78.62%).

### 3.8. Habitat Transition Pathways

**Figure 9:**
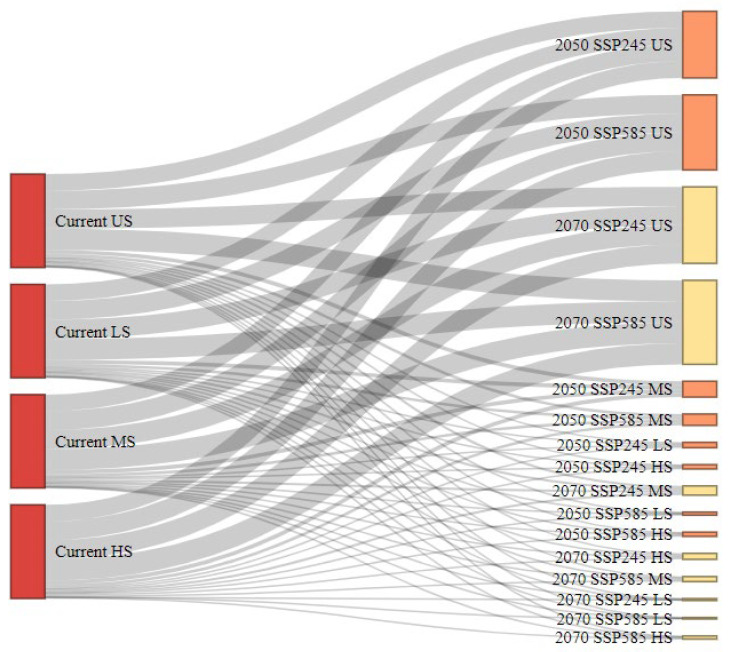
Transitions in *A. dorsata* habitat suitability from current conditions to future projections under mid-century (2050) and late-century (2070) climate scenarios (SSP245 and SSP585). Unsuitable (US), less suitable (LS), moderately suitable (MS), and highly suitable (HS).

The Sankey diagram comprehensively visualizes habitat suitability transitions for *A. dorsata* from the current distribution to future climate projections under SSP245 and SSP585 scenarios (Figure 9). The diagram captures the flow of habitat categories including unsuitable (US), less suitable (LS), moderately suitable (MS), and highly suitable (HS) across different periods, including mid-century (2050) and late-century (2070). Notably, a substantial shift towards increased “Unsuitable” habitats is evident, particularly pronounced under the SSP585 pathway for late-century. This trend suggests significant habitat loss due to the intensifying impacts of climate change. The flow lines demonstrate that current “Highly Suitable” and “Moderately Suitable” areas predominantly transition to lower suitability categories or become “Unsuitable” over time. This indicates a reduction in favorable habitat, reflecting habitat degradation across future scenarios. The pattern also showed variability between SSP245 and SSP585, with SSP585 displaying more severe transitions, underscoring the increased risk associated with higher emission pathways. Overall, this visualization highlights the vulnerability of *A. dorsata* habitats in Pakistan under projected climate change, emphasizing the potential impact on the species’ future distribution.

## 4. Discussion

We used MaxEnt modeling to assess the current and future habitat suitability and distributions of *A. dorsata* across Pakistan. The MaxEnt approach is considered highly reliable and accepted for ecological niche modeling [64,126]. It is known for and is widely recognized for its robust predictive performance with complex interactions between species occurrences and environmental factors [145]. We found that MaxEnt performed exceptionally well in our study across all metrics, with an AUC of 0.91, which is comparable to other studies that used MaxEnt for similar ecological assessments [11,146]. In addition, performing MaxEnt in R improved the overall model-building process with features including seamless data handling, reproducibility, and customization [147]. Environmental data are processed, models are trained, and they are evaluated with R packages like *dismo* and *raster*, making the workflow efficient and scalable for our research objectives and conservation needs [147]. These advantages underscore the utility of MaxEnt for ecological modeling and demonstrate the further convenience of running habitat suitability models in R, simplifying analyses and increasing clarity of research outputs [138]. Based on the good AUC values and model fit of this study, MaxEnt should be considered as a primary tool for species distribution modeling and conservation planning [148].

Our MaxEnt model generated response curves for the selected eight variables, allowing us to understand how climatic variables influence the suitability distribution of *A. dorsata*. Results showed habitat suitability for *A. dorsata* to increase under moderate temperature and precipitation values but to decrease when variables are extreme. Like other bee species, *A. dorsata* is highly sensitive to temperature and moisture fluctuations, which impact its habitat, foraging, reproduction, and ultimate survival [149,150]. Annual mean temperature (Bio1) and temperature seasonality (Bio4) also showed an influence on the species’ suitable habitat. Optimal foraging and hive health are essential to *A. dorsata* sustainability, and these are best achieved under moderate temperature conditions [151]. Extreme temperatures can compromise brood development and honey production in *A. dorsata* colonies [152]. Numerous studies highlight temperature as a key factor in bees’ distribution and habitat, with species typically adapted to temperature ranges that support optimal physiological performance and energy efficiency [153].

Precipitation, particularly during the wettest (Bio8) and coldest quarters (Bio18), emerged as a crucial factor influencing the habitat suitability of *A. dorsata*. The fact reflects the species’ dependence on water availability to sustain nectar- and pollen-producing flora essential for foraging, highlighting the interplay between hydrological cycles and floral resource availability. Floral availability and diversity, critical for sustaining bee populations, are closely tied to moderate rainfall levels [154]. Excessive or insufficient rainfall reduces nectar and pollen availability or limits access, making habitats suitable for the bee species [153,155]. The response curves indicate that *A. dorsata* favors stable rainfall patterns, adopting best to environments with consistent water availability. This finding aligns with the previous studies on pollinators, which highlight the importance of balanced precipitation in sustaining plant communities that provide nectar and pollen [156]. *A. dorsata* exhibits a nuanced adaptation to temperature and precipitation, balancing resource competition with avoidance of climatic extremes that could endanger habitat, colony health, and productivity [157,158]. Vegetation, flowering patterns, and abundance of pollinator communities like bees are intricately tied to specific temperatures and precipitation levels. High temperatures accelerate evapotranspiration, often leading to dry soil conditions that stress vegetation and reduce nectar and pollen availability in flowering plants [156]. Thermal stress can also disrupt flowering cycles, creating a mismatch between peak flowering and periods when bees rely most on floral resources [159,160].

The projected habitat loss and shift of *A. dorsata* in Pakistan could be attributed to the country’s significant environmental challenges they are facing due to climate change and socioeconomic pressures. Key drivers include projected temperature increase and changes in precipitation patterns and intensities, both of which affect the floral resources and composition and ecological stability that *A. dorsata* depends on [153,156]. As a species adapted to tropical climates with moderate temperature and rainfall, *A. dorsata* is particularly susceptible to the expected rise in temperature in Pakistan, which studies indicate may outpace the global average [161,162]. Under high-emission scenarios, habitat loss becomes even more pronounced due to intensified heat waves, droughts, and erratic rainfalls [163]. These extreme conditions could disrupt the flowering cycles and decrease the availability of essential foraging plants, ultimately threatening the survival and reproductive success of *Apis dorsata*. The species’ dependence on a variety of floral resources heightens its vulnerability to fluctuations in plant diversity and abundance, which are influenced by temperature and precipitation instability [154]. Rising temperatures can also directly impact *A. dorsata* by altering both behavior and physiology, resulting in constrained foraging activity and diminished colony productivity. Studies concluded that bees reduce their foraging activity at temperatures exceeding their optimal range, which could intensify resource shortages during critical times [164,165,166]. In addition to climatic changes, shafting land use patterns in Pakistan present an additional threat to *A. dorsata* habitats. Rapid population growth and associated rise in urban development and expansion of agricultural lands are leading to fragmentation and reduction in natural habitats [167]. This shrunk the foraging landscape for the species and exposed it to anthropogenic pressures. Additionally, the rise in agricultural land caused a rise in the use of pesticides [153].

Under future climatic conditions, the *A. dorsata* habitats in Pakistan are likely to shift towards higher altitudes or more northern regions, a trend consistent with the global observation of species moving to cooler or more favorable environments as a response to global warming [1]. Shifts towards northern, higher-altitude regions, including Khyber Pakhtunkhwa and the Himalayan foothills, are expected to provide cooler, more stable habitats with fewer disturbances [168,169]. Such areas are predicted to maintain more stable floral resources, offering potential refuges to the species [153]. Additionally, these areas have reduced human disturbances compared to lower land area of the country. However, such habitat shifts are often faced with geographical and ecological constraints [170,171], especially in the case of areas like Pakistan, having a diverse landscape.

Pakistan is recognized as one of the top five countries most affected by climate change, experiencing frequent and severe climate anomalies, including intense floods, heat waves, and irregular heavy rainfall [172]. These extreme weather events have profound implications for both biodiversity and human livelihoods and are projected to escalate in frequency and severity if current climate trends persist [173]. Our study’s future projections for *A. dorsata* habitat suitability highlight a significant decline in areas deemed moderately and highly suitable for the species. This anticipated loss aligns with the broader impacts of climate change on ecosystems across Pakistan and poses a substantial risk to pollinators and the services they provide.

The plains area, especially in Punjab, is expected to experience significant warming, which could disrupt the *A. dorsata* habitat, forging activity, and floral resource availability. Additionally, temperature fluctuations and altered rainfall patterns adversely impact vegetation, reducing the abundance of flowering plants essential to *A. dorsata* [174]. This reflects findings in similar studies, where climatic variability has been shown to disrupt pollinator habitats, reduce floral resources, and alter species distributions [175,176]. In regions like Punjab and Sindh, where human population density and land use change are prominent, the compounded effects of habitat fragmentation and environmental stressors further exacerbate the loss of viable habitats for *A. dorsata*. The significant loss of suitable habitat for *A. dorsata* in Punjab’s lower plains could also be attributed to the excessive use of pesticides in extensive farming. As Pakistan’s agricultural hub, Punjab heavily relies on agrochemicals to maximize crop yield [177,178]. Pesticides harm bee populations both directly and indirectly [179,180].

### Conservation and Management Strategies

To address the urgent challenges facing *A. dorsata* and their habitat in Pakistan, a comprehensive, multi-faceted conservation strategy is essential. Key recommendations include promoting sustainable agriculture practices like integrated pest management (IPM) and agroecology (e.g., crop rotation, intercropping) to curb pesticide use while fostering pollinator-friendly environments. Reforestation and afforestation efforts should prioritize native flowering plants, enhancing foraging and nesting sites while boosting habitat resilience, particularly in northern and central Pakistan. Planting bee-friendly flora in currently suitable areas could provide stable nectar and pollen sources, benefiting *A. dorsata* populations and, ultimately, contributing to climate mitigation by supporting ecosystems that store carbon.

To mitigate climate change impacts, policies focusing on reforestation and carbon sequestration can help stabilize local microclimates, while adaptive strategies like creating artificial nesting sites in affected areas support population stability during environmental shifts. Community engagement is critical, with programs educating locals on the ecological and economic benefits of pollinators and offering sustainable beekeeping as a livelihood. Strengthening regulations to control agrochemical overuse, deforestation, and habitat conversion will reduce habitat degradation and incentivize pollinator-friendly practices among farmers. Finally, establishing long-term monitoring and research programs will support adaptive management, with collaborative research guiding targeted conservation actions. Implementing these strategies can help preserve *A. dorsata* and pollination services, essential for Pakistan’s ecological and agricultural sustainability.

## 5. Conclusions

This study was carried out to identify, categorize, and quantify *A. dorsata* habitat suitability and distribution under current and future climatic conditions in Pakistan, one of the most vulnerable countries to climate change. Our results concluded that the species habitat suitability is highly related to stable and moderate ranges of both temperature and precipitation. A decline in species habitat was observed for extreme climatic conditions. Moreover, the species would lose 80% of its current suitable habitat to climate change under extreme greenhouse emission scenarios. This shows the species’ sensitivity to the changing climatic conditions and global warming. Our model predicted a shift in species habitats towards higher altitudes in the northern parts of Pakistan. To mitigate the impacts of climate change on species habitats, the country government with the help of national and international conservation organizations should design conservation strategies and policies to encounter the climate change effects. This study underscores the importance of adaptive management practices considering dynamic ecological interactions amid escalating climatic challenges.

## Figures and Tables

**Table 1:** Current and future habitat suitability breakdowns and projections for *A. dorsata* under different climate scenarios.

Future Projection		Scenario	Habitat Categories				Total
Model	Year		Unsuitable (km^2^)	Less Suitable (km^2^)	Moderately Suitable (km^2^)	Highly Suitable (km^2^)	
		Current	629,817	51,312	150,975	49,792	881,896
CNRM-CM6-1	2050	SSP245	708,119	31,569	103,135	39,073	881,896
		SSP585	725,579	27,196	93,156	35,965	881,896
	2070	SSP245	741,449	18,162	86,274	36,011	881,896
		SSP585	792,177	14,285	45,691	29,743	881,896
EPI-ESM1-2-HR-1	2050	SSP245	713,598	43,872	96,561	27,865	881,896
		SSP585	737,895	34,916	86,756	22,329	881,896
	2070	SSP245	741,035	29,593	86,721	24,547	881,896
		SSP585	802,169	14,325	54,756	10,646	881,896

**Table 2:** Percentage changes in habitat categories for *A. dorsata* under different climate models (CNRM-CM6-1 and EPI-ESM1-2-HR-1) and scenarios (SSP245 and SSP585) for mid-century (2050) and late-century (2070) projections.

Future Projection	Period	Scenario	Habitat Categories			
Model			US	LS	MS	HS
		Current	629,817	51,312	150,975	49,792
CNRM-CM6-1	2050	SSP245% Change	12	−38	−32	−22
		SSP585% Change	15	−47	−38	−28
	2070	SSP245% Change	18	−65	−43	−28
		SSP585% Change	26	−72	−70	−40
EPI-ESM1-2-HR-1	2050	SSP245% Change	13	−14	−36	−44
		SSP585% Change	17	−32	−43	−55
	2070	SSP245% Change	18	−42	−43	−51
		SSP585% Change	27	−72	−64	−79

Positive values indicate an increase in the unsuitable habitat, while negative values show a decrease in less suitable, moderately suitable, and highly suitable habitats compared to the current distribution. US: Unsuitable, LS: less suitable, MS: moderately suitable, and HS: highly suitable.

## Data Availability

The data will be available on request.

## Supplementary Materials

The following supporting information can be downloaded at https://www.mdpi.com/article/10.3390/insects16030289/s1, Table S1: The literature on different ecological aspects of *A. dorsata* to supplement the occurrence data; Table S2: Environmental variables used in MaxEnt modeling; Figure S1: Correlation among all 19 variables. The red highlight shows the variables having correlation less than 0.75.
