# Supplementary material for: Forecasting the Impact of Climate Change on Apis dorsata (Fabricius, 1793) Habitat and Distribution in Pakistan
Source: Insects. 2025 Mar 11;16(3):289. doi: 10.3390/insects16030289 (PMC11942931; doi:10.3390/insects16030289)
Supplement: Supplementary file 1 [file insects-16-00289-s001.zip › insects-3405022-supplementary.pdf]

Supplementary material S1: Literature on different ecological aspects of Apis dorsata to supplement the occurrence data

Paper Title	Journal	Year
Foraging behavior of the giant honey bee, Apis dorsata F. (Hymenoptera: Apidae) in sunflower (Helianthus annuus L.) at Peshawar District of Pakistan	Pure and Applied Biology	2018
Morphological and genetic characterization of various Apis species captured from selected sites of Punjab	FEB-Fresenius Environmental Bulletin	2022
Physico-chemical and antimicrobial assessment of honey of Apis dorsata from different geographical regions of Pakistan	International Journal of Agricultural Science Research	2014
Bio-chemical analysis of honey made by three Apis florea, Apis mellifera, and Apis dorsata honeybee species from Punjab region	Pure and Applied Biology	2023
Methods to control migration by Apis dorsata colonies in Pakistan	Bee World	1989
Physicochemical variations in the honey produced by Apis dorsata from Punjab, Pakistan	Pure and Applied Biology	2017
Spatiotemporal occurrence of beehives of genus Apis in Northern Punjab and Azad Jammu and Kashmir, Pakistan	Kuwait Journal of Science	2023
Comparison of wild honeybees in the pollination of strawberries in Bahawalpur, Pakistan	Revista de la Sociedad Entomológica Argentina	2023
Foraging behavior of the giant honey bee, *Apis dorsata* F. (Hymenoptera: Apidae) in sunflower (*Helianthus annuus* L.) at Peshawar District of Pakistan	Pakistan Journal of Zoology	2018
Comparison of *Apis cerana*, *Apis dorsata*, *Apis florea*, and *Apis mellifera* honey from different areas of Pakistan	Asian Journal of Experimental Biological Sciences	2015
Genetic analysis of honey bee (*Apis dorsata*) populations using random amplified polymorphic DNA (RAPD) markers	Journal of King Saud University – Science	2020
Detection of insecticide residues in honey of *Apis dorsata* F. from Southern Punjab, Pakistan	Pakistan Journal of Zoology	2017
Antibacterial properties of *Apis dorsata* honey against some bacterial pathogens	Journal of King Saud University – Science	2021
Toxicity of some insecticides to the haemocytes of giant honeybee, *Apis dorsata* F. under laboratory conditions	Journal of Entomology and Zoology Studies	2017
Diversity and relative abundance of pollinator fauna of canola (*Brassica napus* L. Var. Chakwal Sarsoon) with managed *Apis mellifera* L. in Pothwar Region, Gujar Khan, Pakistan	Pakistan Journal of Zoology	2018
Health benefits of honey and ethnobotanical uses of its bee flora from Lakki Marwat district, Khyber Pakhtunkhwa, Pakistan	Journal of Ethnobiology and Ethnomedicine	2021
Yearlong association of Apis dorsata and Apis florea with flowering plants: Planted forest vs. agricultural landscape	Sociobiology	2017
Toxicity of Commonly Used Insecticides against Apis dorsata (Hymenoptera: Apidae) in South Punjab, Pakistan	Not specified (in progress)	2024
Detection of insecticide residues in honey of Apis dorsata F. from Southern Punjab, Pakistan	Pakistan Journal of Zoology	2017
Assessment of physico-chemical and antimicrobial of honey of Apis dorsata from different locations of Pakistan	Global Science Research Journals	2014

Supplementary material S2: Environmental variables used in MaxEnt modeling.

Environmental variable	*Interpretation*	*Source*
bio1	Annual mean temperature	http://www.worldclim.org

bio2	Mean diurnal range (mean of monthly [max temp - min temp])	http://www.worldclim.org
bio3	Isothermality (Bio2/Bio7) (* 100)	http://www.worldclim.org
bio4	Temperature Seasonality (standard deviation *100)	http://www.worldclim.org
bio5	Max Temperature of Warmest Month	http://www.worldclim.org
bio6	Min Temperature of Coldest Month	http://www.worldclim.org
bio7	Temperature Annual Range (Bio5-Bio6)	http://www.worldclim.org
bio8	Mean Temperature of Wettest Quarter	http://www.worldclim.org
bio9	Mean Temperature of Driest Quarter	http://www.worldclim.org
bio10	Mean Temperature of Warmest Quarter	http://www.worldclim.org
bio11	Mean Temperature of Coldest Quarter	http://www.worldclim.org
bio12	Annual precipitation	http://www.worldclim.org
bio13	Precipitation of Wettest Month	http://www.worldclim.org
bio14	Precipitation of driest month	http://www.worldclim.org
bio15	Precipitation Seasonality (Coefficient of Variation)	http://www.worldclim.org
bio16	Precipitation of Wettest Quarter	http://www.worldclim.org
bio17	Precipitation of Driest Quarter	http://www.worldclim.org
bio18	Precipitation of Warmest Quarter	http://www.worldclim.org
bio19	Precipitation of Coldest Quarter	http://www.worldclim.org

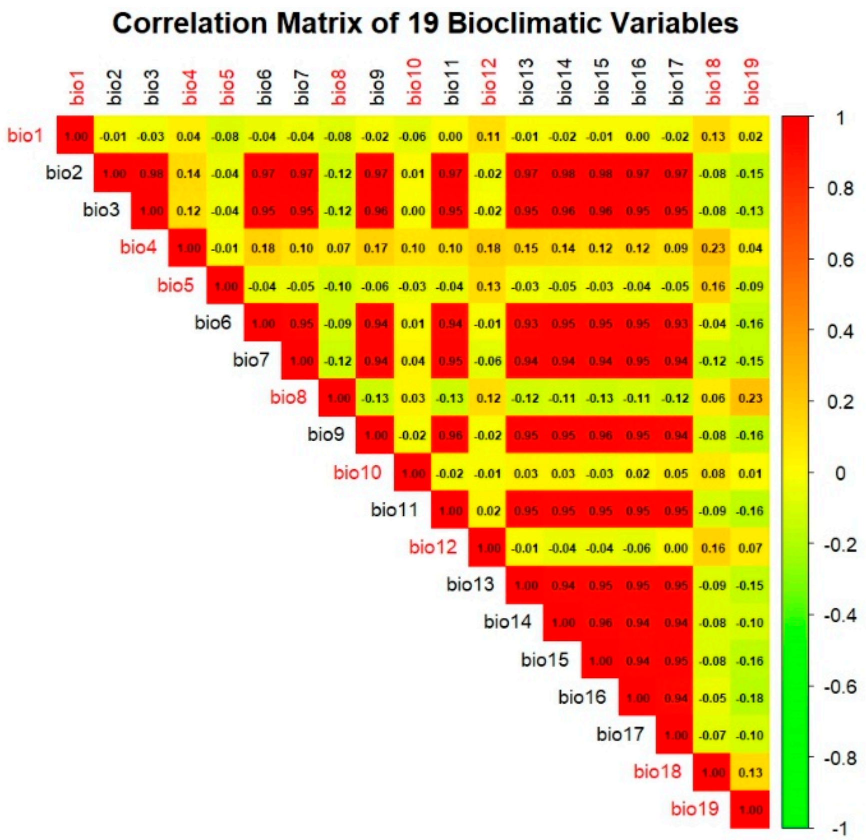

Supplementary material S3: Correlation among all 19 variables. The red highlighted are the variables having correlation less than 0.75.
